# Supplementary material for: Medical Students’ Perspectives on LGBTQI+ Healthcare and Education in Germany: Results of a Nationwide Online Survey
Source: Int J Environ Res Public Health. 2022 Aug 13;19(16):10010. doi: 10.3390/ijerph191610010 (PMC9408586; doi:10.3390/ijerph191610010)
Supplement: Supplementary file 1 [file ijerph-19-10010-s001.zip › ijerph-1825565-supplementary.pdf]

1 Survey instructions and items (translation)

Dear Students,

In the following, we would like to ask you to comment on several statements. These statements relate to LGBTIQ+ individuals, i.e. lesbian, gay, bisexual, trans*, intersex, and queer individuals. The statements come from evidence-based sources and will be posted on our study website (https://psychosomatik.owl.rub.de/) after the research is completed. We also ask you to provide some statements about yourself, such as age, gender, semester, university, and also whether you define yourself as part of the LGBTIQ+ community. All information is anonymized and you will help us to evaluate the current content of the medical school. Thank you for your participation!

Knowledge

For each statement, please rate whether the statement is correct ('Yes') or not ('No').

		**yes**	**no**
1	The physical sex of trans*persons can be clearly assigned, but this is not perceived as correct by them.	X	
2	Many people have intimate or sexual contacts with persons of the same sex without identifying as homosexual.	X	
3	Homosexual and bisexual men, are more frequently victims of physical violence than average.	X	
4	Gay men earn significantly lower wages than heterosexual men, which can lead to fewer financial resources and poverty in old age over a lifetime.	X	
5	For LGBTIQ+ people, access to adequate medical treatment is more difficult.	X	
6	The percentage of people in the general population who identify as not exclusively heterosexual is 3-10%.	X	
7	Heterosexual women are more likely than heterosexual men to hold hostile attitudes toward gay people.		X
8	Intersex people are those whose physical sex cannot be clearly assigned.	X	
9	Individuals' gender identity, sexual orientation, and sexual behavior can change over the lifespan.	X	
10	Suicide rates among LGBTIQ+ persons are higher than average.	X	
11	LGBTIQ+ individuals are more likely than average to exhibit anxiety disorders and depression.	X	
12	It is possible to treat depression and continue gender reassignment at the same time.	X	
13	A trans* patient with a history of schizophrenia may not implement gender reassignment.		X
14	There are a large number of randomized clinical trials on LGBTIQ+ individuals.		X

15	In most cases, homosexual individuals are dissatisfied with their gender assignment.		X
16	Trans* women have a higher risk of developing cerebral ischemia compared to biological women of the same age.	X	
17	Gender reassignment surgery can be performed well into old age.	X	
18	A trans* woman can no longer have an orgasm after gender reassignment surgery.		X
19	Many heterosexual men and women report having homosexual fantasies as well.	X	

Prejudice

For each statement, please rate whether the statement is correct ('Yes') or not ('No').

		**yes**	**no**
1	The majority of people who identify as homosexual have experienced abuse by persons of the same sex in childhood or adolescence.		X
2	Trans identity and pedophilia are related.		X
3	Trans* persons develop their identity through a false upbringing in the parental home.		X
4	Biological males who dress feminine are always homosexual.		X
5	Due to gender-sensitive education in school, the number of trans* children is rapidly increasing.		X
6	Most homosexual men and women want to be heterosexual.		X
7	Most homosexuals want to encourage or entice others to live homosexually as well.		X
8	Because of the positive portrayal of homosexuality in the media, more and more young people are choosing to become homosexual.		X
9	Contact with homosexual teachers causes psychological harm to children in their contact behavior and sexual development.		X

Contact

For each statement, please rate whether the statement applies to you.

		**yes**	**no**
1	In my everyday life, I have regular contact with at least one LGBTIQ+ person.	□	□
2	A family member close to me openly identifies as homosexual or bisexual.	□	□
3	A family member close to me openly identifies as trans*.	□	□

4	I am friends with a person who openly identifies as gay or bisexual.	□	□
5	I am friends with a person who openly identifies as trans*.	□	□
6	I define myself as part of the LGBTIQ+ community.	□	□

Comfort

	**I feel comfortable …**	**yes**	**Rather yes**	**Rather no**	**no**
1	taking a sexual history from a heterosexual person who is the same sex as me.	□	□	□	□
2	taking a sexual history from a heterosexual person who is of the opposite sex to me.	□	□	□	□
3	taking a sexual history from a homosexual or bisexual person who is the same sex as me.	□	□	□	□
4	taking a sexual history from a homosexual or bisexual person who is of a different sex than I am.	□	□	□	□
5	taking a sexual history from a trans* person.	□	□	□	□
6	performing a physical examination on a heterosexual person who is the same sex as me.	□	□	□	□
7	performing a physical examination on a heterosexual person who is of a different sex than me.	□	□	□	□
8	performing a physical examination on a homosexual or bisexual person who is the same sex as me.	□	□	□	□
9	performing a physical examination on a homosexual or bisexual person who is of a different sex than me.	□	□	□	□
10	performing a physical examination on a trans* person.	□	□	□	□
11	being medically trained by a homosexual or bisexual person.	□	□	□	□
12	being medically trained by a trans* person.	□	□	□	□
13	when a man and a woman show their affection to each other in public, e.g. by kissing.	□	□	□	□
14	when two women show their affection to each other in public, e.g. by kissing.	□	□	□	□
15	when two men show their affection to each other in public, e.g. by kissing.	□	□	□	□

Efficacy Beliefs

For each statement, please rate whether the statement applies to you.

I am confident that...	**yes**	**Rather yes**	**Rather no**	**no**

1	... the study of medicine contributes to recognizing and addressing the needs of LGBTIQ+ persons and special requirements in their medical care.	□	□	□	□
2	... based on the knowledge I have acquired so far, I am able to take a comprehensive sexual history of LGBTIQ+ persons.	□	□	□	□
3	... LGBTIQ+ persons in Germany receive adequate medical assistance without any problems or restrictions.	□	□	□	□

Teaching

The following statements always refer to the course content of the university at which you are currently enrolled. Please rate for each section of study that applies to you and omit those that do not.

		**yes**	**Rather yes**	**Rather no**	**no**
1	In the course of the basic studies ("pre-clinic") LGBTIQ+ topics were covered.	□	□	□	□
2	In the course of the main studies ("clinic") LGBTIQ+ topics were covered.	□	□	□	□
3	In the course of the practical year ("PJ") LGBTIQ+ topics were covered.	□	□	□	□
4	I consider LGBTIQ+ related teaching in the medical curriculum necessary and important.	□	□	□	□
5	I would like to see more teaching in the medical curriculum on LGBTIQ+ related aspects.	□	□	□	□

2 Survey instructions and items (original)

Liebe Studierende,

Im Folgenden möchten wir Sie bitten, zu mehreren Aussagen Stellung zu beziehen. Diese Aussagen beziehen sich auf LSBTIQ+ Personen, d.h. lesben, schwule, bisexuelle, trans*, intergeschlechtliche und queere Personen. Die Aussagen entstammen evidenzbasierten Quellen und werden nach Abschluss der Untersuchung auf unserer Studien-Website (https://psychosomatik.owl.rub.de/) veröffentlicht. Außerdem bitten wir Sie um einige Aussagen zu Ihrer Person, z.B. Alter, Geschlecht, Semester, Universität, und auch, ob Sie sich selbst als Teil der LSBTIQ+ Community definieren. Alle Angaben sind **anonymisiert** und Sie helfen uns damit, die aktuellen Inhalte des Medizinstudiums zu evaluieren. Vielen Dank für Ihre Teilnahme!

Wissen

Bitte bewerten Sie für jede einzelne Aussage, ob die Aussage korrekt ist ('Ja') oder nicht ('Nein').

		**ja**	**nein**
1	Trans*Personen sind diejenigen, deren körperliches Geschlecht eindeutig zugeordnet werden kann, dieses aber nicht als richtig empfunden wird.	X	
2	Viele Menschen haben intime bzw. sexuelle Kontakte mit Personen des gleichen Geschlechts, ohne sich als homosexuell zu identifizieren.	X	
3	Homo- und bisexuelle Männer, sind überdurchschnittlich oft Opfer von körperlicher Gewalt.	X	
4	Schwule Männer erzielen signifikant geringere Löhne als heterosexuelle Männer, was über die Lebenszeit zu geringeren finanziellen Ressourcen und Altersarmut führen kann.	X	
5	Der Zugang zu adäquater medizinischer Behandlung ist für LSBTIQ+ Personen erschwert.	X	
6	Der Anteil von Menschen in der Allgemeinbevölkerung, die sich als nicht ausschließlich heterosexuell bezeichnen liegt bei 3-10%.	X	
7	Heterosexuelle Frauen neigen häufiger als heterosexuelle Männer zu feindseligen Einstellungen gegenüber homosexuellen Personen.		X
8	Intergeschlechtliche Menschen sind diejenigen, deren körperliches Geschlecht nicht eindeutig zugeordnet werden kann.	X	
9	Die geschlechtliche Identität, sexuelle Orientierung und das sexuelle Verhalten von Personen können sich über die Lebenszeit hinweg verändern.	X	
10	Die Suizidrate unter LSBTIQ+ Personen ist überdurchschnittlich hoch.	X	
11	LSBTIQ+ Personen zeigen überdurchschnittlich häufig Angststörungen und Depressionen.	X	
12	Es ist möglich, eine Depression zu behandeln und gleichzeitig eine Geschlechtsangleichung fortzusetzen.	X	

13	Ein(e) Patient*in mit Schizophrenie in der Anamnese darf keine Geschlechtsangleichung umsetzen.		X
14	Es gibt eine große Anzahl an randomisierten klinischen Studien für LSBTIQ+ Personen.		X
15	Homosexuelle Personen sind in den meisten Fällen unzufrieden mit ihrer geschlechtlichen Zuordnung.		X
16	Trans*Frauen haben ein höheres Risiko für das Auftreten von zerebralen Ischämien im Vergleich zu biologischen Frauen des gleichen Alters.	X	
17	Geschlechtsangleichende Operationen können bis ins höhere Alter durchgeführt werden.	X	
18	Eine Trans*Frau kann nach einer geschlechtsangleichenden Operation keinen Orgasmus mehr bekommen.		X
19	Viele heterosexuelle Männer und Frauen berichten davon, auch homosexuelle Fantasien zu haben.	X	

Vorurteile

Bitte bewerten Sie für jede einzelne Aussage, ob die Aussage korrekt ist ('Ja') oder nicht ('Nein').

		**ja**	**nein**
1	Die Mehrheit der Menschen, die sich als homosexuell identifizieren, haben Missbrauchserfahrungen durch Personen des gleichen Geschlechts in der Kindheit oder Adoleszenz erlebt.		X
2	Transidentität und Pädophilie stehen in Zusammenhang miteinander.		X
3	Trans*Personen entwickeln Ihre Identität durch eine falsche Erziehung im Elternhaus.		X
4	Biologische Männer, die sich weiblich kleiden, sind immer homosexuell.		X
5	Durch geschlechtersensible Erziehung in der Schule nimmt die Anzahl an Trans*Kindern rasant zu.		X
6	Die meisten homosexuellen Männer und Frauen wollen heterosexuell sein.		X
7	Die meisten Homosexuellen wollen andere Menschen ermutigen oder dazu verleiten, auch homosexuell zu leben.		X
8	Durch die positive Darstellung von Homosexualität in den Medien, entscheiden sich immer mehr Jugendliche, homosexuell zu werden.		X
9	Der Kontakt mit homosexuellen Lehrkräften fügt Kindern in ihrem Kontaktverhalten und ihrer sexuellen Entwicklung psychologischen Schaden zu.		X

Kontakt

Bitte bewerten Sie für jede einzelne Aussage, ob die Aussage auf Sie zutrifft.

		ja	nein
1	In meinem Alltag habe ich regelmäßig Kontakt zu mindestens einer LSBTIQ+ Person.	□	□
2	Ein Familienmitglied, das mir nahesteht, identifiziert sich offen als homo- oder bisexuell.	□	□
3	Ein Familienmitglied, das mir nahesteht, identifiziert sich offen als trans*.	□	□
4	Ich bin mit einer Person befreundet, die sich offen als homo- oder bisexuell identifiziert.	□	□
5	Ich bin mit einer Person befreundet, die sich offen als trans* identifiziert.	□	□
6	Ich definiere mich selbst als Teil der LSBTIQ+ Community	□	□

Komfort

	**Ich fühle mich wohl, …**	**ja**	**eher ja**	**eher nein**	**nein**
1	eine Sexualanamnese bei einer heterosexuellen Person, die das gleiche Geschlecht hat wie ich, zu erheben.	□	□	□	□
2	eine Sexualanamnese bei einer heterosexuellen Person, die anderen Geschlechts ist als ich, zu erheben.	□	□	□	□
3	eine Sexualanamnese bei einer homo- oder bisexuellen Person, die das gleiche Geschlecht hat wie ich, zu erheben.	□	□	□	□
4	eine Sexualanamnese bei einer homo- oder bisexuellen Person, die anderen Geschlechts ist als ich, zu erheben.	□	□	□	□
5	eine Sexualanamnese bei einer trans*Person zu erheben.	□	□	□	□
6	eine körperliche Untersuchung bei einer heterosexuellen Person, die das gleiche Geschlecht hat wie ich, durchzuführen.	□	□	□	□
7	eine körperliche Untersuchung bei einer heterosexuellen Person, die anderen Geschlechts ist als ich, durchzuführen.	□	□	□	□
8	eine körperliche Untersuchung bei einer homo- oder bisexuellen Person, die das gleiche Geschlecht hat wie ich, durchzuführen.	□	□	□	□
9	eine körperliche Untersuchung bei einer homo- oder bisexuellen Person, die anderen Geschlechts ist als ich, durchzuführen.	□	□	□	□
10	eine körperliche Untersuchung bei einer trans*Person durchzuführen.	□	□	□	□
11	von einer homo- oder bisexuellen Person medizinisch ausgebildet zu werden.	□	□	□	□
12	von einer trans*Person medizinisch ausgebildet zu werden.	□	□	□	□
13	wenn sich ein Mann und eine Frau ihre Zuneigung in der Öffentlichkeit zeigen, z.B. indem sie sich küssen.	□	□	□	□
14	wenn sich zwei Frauen ihre Zuneigung in der Öffentlichkeit zeigen, z.B. indem sie sich küssen.	□	□	□	□

15	wenn sich zwei Männer ihre Zuneigung in der Öffentlichkeit zeigen, z.B. indem sie sich küssen.	□	□	□	□

Überzeugungen

Bitte bewerten Sie für jede einzelne Aussage, inwiefern die Aussage auf Sie zutrifft.

	**Ich bin davon überzeugt, dass…**	**ja**	**eher ja**	**eher nein**	**nein**
1	… das Medizinstudium dazu beiträgt, die Bedürfnisse von LSBTIQ+ Personen und spezielle Anforderungen in deren medizinischen Versorgung zu erkennen und zu adressieren.	□	□	□	□
2	… ich auf Basis bisher erworbenen Wissens in der Lage bin, eine umfassende Sexualanamnese bei LSBTIQ+ Personen zu erheben.	□	□	□	□
3	… LSBTIQ+ Personen in Deutschland problemlos und uneingeschränkt adäquate medizinische Hilfe bekommen.	□	□	□	□

Lehre

Die folgenden Aussagen beziehen sich immer auf die Lehrinhalte der Universität, an der Sie aktuell immatrikuliert sind. Bitte bewerten Sie für jeden Studienabschnitt, der auf Sie zutrifft und lassen Sie nicht zutreffende aus.

		**ja**	**eher ja**	**eher nein**	**nein**
1	Im Verlauf des Grundstudiums („Vorklinik“) wurden LSBTIQ+ Themen behandelt.	□	□	□	□
2	Im Verlauf des Hauptstudiums („Klinik“) wurden LSBTIQ+ Themen behandelt.	□	□	□	□
3	Im Verlauf des Praktischen Jahres („PJ“) wurden LSBTIQ+ Themen behandelt.	□	□	□	□
4	Ich halte LSBTIQ+ bezogene Lehre im medizinischen Curriculum für notwendig und wichtig	□	□	□	□
5	Ich würde mir mehr Lehre im medizinischen Curriculum zu LSBTIQ+ bezogenen Aspekten wünschen	□	□	□	□

3 Additional results

Table S1. *Means, standard deviations, and correlations with confidence intervals*

Variable	*M*	*SD*	1	2	3	4	5	6
1. Knowledge	15.31	1.91						
2. Prejudice	0.37	0.98	-.36**<br>[-.42, -.29]					
3. Contact	2.12	1.05	.23**<br>[.16, .30]	-.16**<br>[-.24, -.09]				
4. Comfort	50.34	5.79	.17**<br>[.10, .24]	-.25**<br>[-.32, -.17]	.22**<br>[.15, .29]			
5. Efficacy beliefs	5.89	1.99	-.25**<br>[-.32, -.18]	.25**<br>[.18, .32]	-.06<br>[-.13, .02]	.02<br>[-.06, .09]		
6. Imp. of teaching	7.04	1.62	.37**<br>[.30, .44]	-.47**<br>[-.53, -.41]	.27**<br>[.20, .34]	.23**<br>[.16, .30]	-.37**<br>[-.44, -.30]	
7. Exp. teaching	1.61	0.62	-.08*<br>[-.16, -.00]	.07<br>[-.01, .14]	.06<br>[-.02, .13]	-.05<br>[-.13, .03]	.36**<br>[.29, .42]	-.11**<br>[-.19, -.04]

Note. M and *SD* are used to represent mean and standard deviation, respectively. Values in square brackets indicate the 95% confidence interval for each correlation. * indicates $p < .05$. ** indicates $p < .01$.

Pearson's rank correlations were computed to assess the pairwise relationships between knowledge, prejudice, contact, comfort, efficacy beliefs, experienced teaching and importance of teaching (Table S1). Only significant correlations greater than .3 or lower than -.3 are reported below.

There were weak positive correlations between knowledge and prejudice (r(657) = -.36, p < .001). Knowledge and importance of teaching were positively correlated (r(647) = .37, p < .001). Importance of teaching and prejudice (r(647) = -.47, p < .001) as well as importance of teaching and efficacy beliefs (r(647) = -.37, p < .001) were correlated negatively. We found a positive weak correlation between experienced teaching and efficacy beliefs (r(641) = .36, p < .001).
